# Capacitive storage at nitrogen doped amorphous carbon electrodes: structural and chemical effects of nitrogen incorporation†

**DOI:** 10.1039/c8ra10187f

**Published:** 2019-01-30

**Authors:** Md. Khairul Hoque, James A. Behan, Serban N. Stamatin, Federico Zen, Tatiana S. Perova, Paula E. Colavita

**Affiliations:** School of Chemistry, CRANN, AMBER Research Centres, Trinity College Dublin Dublin 2 Ireland colavitp@tcd.ie; University of Bucharest, Faculty of Physics, 3Nano-SAE Research Centre 405 Atomistilor Str, Magurele 077125 Bucharest Romania; Department of Electronic and Electrical Engineering, Trinity College Dublin Dublin 2 Ireland; ITMO University 49 Kronverskiy pr. Saint Petersburg 197101 Russia

## Abstract

Nitrogen incorporated carbon materials play an important role in electrochemical energy conversion technologies from fuel cells to capacitive storage devices. This work investigates the effects of nitrogen incorporation on capacitance, work function and semiconductor properties of amorphous carbon thin film electrodes. Nitrogenated electrodes (a-C:N) electrodes were synthesized *via* magnetron sputtering and characterized using X-ray photoelectron spectroscopy, ultraviolet photoelectron spectroscopy (UPS), Raman spectroscopy, cyclic voltammetry (CV), and electrochemical impedance spectroscopy (EIS). EIS was carried in both aqueous (0.1 M KCl) and organic (0.1 M TBAPF_6_/acetonitrile) electrolytes to discriminate between pseudocapacitive contributions and changes to semiconductor properties of the materials arising from structural and chemical disruption of the graphitic carbon scaffold. Raman and UPS spectroscopy both suggest that nitrogen incorporation increases the metallic character of the disordered carbon matrix at low-intermediate concentrations, whereas further nitrogen incorporation results in significantly more defective carbon with small graphitic cluster size. EIS studies in 0.1 M KCl indicate that the capacitance of a-C:N electrodes increases relative to nitrogen-free a-C electrodes due to a combination of microroughness and pseudocapacitive contributions in parallel to those of the double layer capacitance. Results in 0.1 M TBAPF_6_ in acetonitrile which are dominated by the interfacial capacitance, show that initial nitrogen incorporation into the disordered carbon scaffold compensates for p-type properties in the disordered carbon matrix, resulting in an increase in metallic character. Greater levels of nitrogenation, are instead disruptive and increase defect density while decreasing the double layer capacitance.

## Abstract 2

Nitrogen incorporation into carbon increases metallic character and capacitance, however high concentrations are instead disruptive and decrease interfacial capacitance.

## Graphical abstract

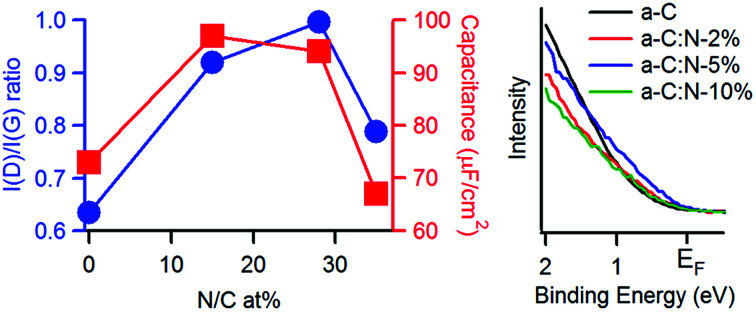

## 1. Introduction

Carbon materials and nanomaterials play an important role in electrochemical energy conversion technologies and are envisioned to remain important for our ability to transition to a more sustainable energy economy. Carbon is ubiquitous as an electrode material and its electronic, surface chemistry and capacitive properties are critical for the design of electrodes in a variety of applications, from fuel cells to capacitive storage devices. Highly graphitic forms of carbon such as carbon blacks and graphite powders are typically used for energy applications due a combination of low cost, good conductivity and reasonable resistance to corrosion. Furthermore, successful development of porous carbon structures has enabled applications in *e.g.* fuel cell electrocatalysis and supercapacitors due to their high specific surface areas and high density of reactive edge sites.

Some of the best performing carbon materials for electrochemical capacitive storage consist of carbon with low or no long-range order.^1^ Nanostructured carbons such as activated carbon, alone or in combination with carbon blacks can display excellent capacitive properties,^2^ that can be comparable to those of more costly “ordered” materials such as graphene or nanotubes.^1^ A high proportion of amorphous regions in these carbon materials enables the development of a pore structure and the presence of high-energy sites resulting from bond distortions and crystallite boundaries. However, the presence of disorder can also result in an undesirable reduction in conductivity and there is consequently great interest in understanding how best to tailor the interplay between interfacial capacitance, surface functionalities and bulk electronic properties in amorphous carbon materials.^1,3^

In this work we focus on a study of the effect of nitrogen incorporation on the capacitive properties of amorphous carbon (a-C) thin-film electrodes. Incorporation of nitrogen functional sites has emerged as one of the important tools available to modulate both electronic and interfacial chemistry of carbon electrodes. Nitrogenation has complex and multifaceted effects on the physico-chemical properties of carbons, resulting in changes in metallic/semiconducting character,^4^ surface free energy and wettability,^5,6^ type of reactive sites and Lewis acid/base behaviour.^7,8^ The effect of nitrogenation on the electrochemical double-layer and redox-capacitance of carbons and nanocarbons has therefore received considerable attention with the objective of designing improved energy storage devices.^9,10^ For instance, the effects of nitrogenation on the capacitance of carbon nanomaterials with long-range order such as carbon nanotubes^11,12^ and graphite^13^ have been studied by various groups.

Recent work from our group studied the effects that heteroatom incorporation has on the kinetics of interfacial charge-transfer at nitrogenated amorphous carbon (a-C:N) electrodes; our work examined to what extent the electrochemical redox response is correlated to the bulk electronic properties or is instead dominated by surface effects.^14^ Herein, we study the effect of N-site incorporation on the organisation of the carbon scaffold and how this determines the capacitive properties of a-C:N electrodes as a result of both surface chemical and electronic effects. a-C:N film electrodes were synthesized with varying nitrogen content *via* DC magnetron sputtering and the materials were characterized using X-ray photoelectron spectroscopy (XPS), ultraviolet photoelectron spectroscopy (UPS), Raman spectroscopy, cyclic voltammetry (CV), and electrochemical impedance spectroscopy (EIS). EIS in both aqueous and organic media enabled the discrimination of electronic and pseudocapacitive contributions to the overall response. Our results show that n-doping can be achieved even with modest additions of N_2_ during carbon deposition; greater concentrations in the deposition gas contribute mostly to the formation of functional groups and the disruption of the carbon scaffold with consequent loss of metallic character.

## 2. Materials and methods

Tetrabutylammonium hexafluorophosphate (TBAPF_6_) (≥99.0%, electrochemical analysis), acetonitrile (MeCN, 99.8%, anhydrous), potassium chloride (Bioxtra, >99.0%), sulfuric acid (95–97%), hydrogen peroxide (>30% w/v), lithium chloride (>99%) were purchased from Sigma Aldrich. Glassy carbon (GC) discs (HTW Sigradur® radius 2.5 mm) and B-doped Si wafers (MicroChemicals; resistivity 5–10 Ω cm) were used as substrates for carbon deposition.

GC disks were polished first with 1200 grit sandpaper, then with 1 μm and 0.3 μm alumina slurries (Buehler) on nylon paper; disks were subsequently polished to a mirror finish using 0.3 and 0.05 μm slurries on MicroCloths® pads (Buehler). Clean disks were mounted in a custom-made Teflon® holder and placed in the vacuum chamber for deposition of thin film carbon electrodes on their surfaces. Si wafers were cleaned with piranha solution (3 : 1H_2_SO_4_ : H_2_O_2_; CAUTION piranha solutions are explosive in contact with organics), rinsed with plenty of Millipore water and dried with Ar prior to deposition. Amorphous carbon films were obtained *via* DC-magnetron sputtering (Torr International) from a graphite target (99.999%) at base pressures <2 × 10^−6^ mbar, deposition pressures 2–7 × 10^−3^ mbar and total gas flow of 50 mL min^−1^, following previously reported protocols.^14,15^ Briefly, nitrogenated amorphous carbon films (a-C:N) were obtained by using N_2_/Ar gas at flow ratios of 2%, 5% and 10% while keeping deposition time constant at 40 min, whereas non-nitrogenated amorphous carbon (a-C) was obtained by carrying out the deposition using 100% Ar during deposition. Electrodes thus deposited are topographically smooth; the mean thickness was determined *via* spectroscopic ellipsometry, following methods detailed in our previous work,^14,16,17^ to be 74, 83, 114 and 123 nm for a-C, a-C:N-2%, 5% and 10%, respectively (see ESI†).

XPS and UPS measurements of a-C and a-C:N films were performed in an Omicron system at 1 × 10^−10^ mbar base pressure, using monochromatic Al Kα source (1486.6 eV) and equipped a multichannel array detector. XPS spectra were collected at 45° take-off angle and 0.5 eV resolution. Peaks were fitted with Voigt functions after Shirley background subtraction using commercial software (CasaXPS); at% composition was obtained from peak area ratios after correction by Scofield relative sensitivity factors (C = 1.0, N = 1.8, O = 2.93). UPS spectra were collected using He(i) excitation source (21.22 eV) at 90° take-off, with 0.02 eV analyser resolution. Negative bias were applied to the sample (0–12 V) to measure the high binding energy edge of the photoelectron spectrum; spectra were then corrected to account for bias and referenced to the Fermi energy measured on a Ag surface in contact with the carbon.^18^ Work function (*ϕ*) values were calculated using the intercept at the binding energy axis of linear fits of the cut-off edge, as *ϕ* = 21.22 – intercept. Raman spectra were measured in backscattering configuration using a Renishaw 1000 micro-Raman system equipped with an Ar^+^ laser for 488 nm excitation and a HeNe laser for 633 nm excitation. The incident beam was focused by a Leica microscope with a 50× magnification objective and short-focus working distance; incident power was kept <2 mW to avoid sample damage. Spectra were baseline corrected using commercial software prior to analysis (Wire 3.2).

Electrochemical measurements were carried using a three-electrode cell controlled by a potentiostat with a graphite rod as counter electrode and Ag/AgCl (sat.) and Ag/Ag^+^ reference electrodes (IJCambria) for characterisation in aqueous and organic electrolyte, respectively. The Ag/Ag^+^ reference 1.0 mM AgNO_3_ in 0.1 M TBAPF_6_ in acetonitrile yielded *E*^0^′ = 0.080 V for 0.001 M Fc/Fc^+^ in the same electrolyte, thus placing the Ag/Ag^+^ potential at 0.320 V *vs.* SHE.^19^ A Teflon static disk holder (Pine Instruments) enclosing a GC disk coated with the sputtered carbon film was used as a working electrode; all contacts were confirmed to be ohmic with <8 Ω resistance. Cyclic voltammetry was carried out in aqueous 0.1 M KCl solutions and in 0.1 M TBAPF_6_ solutions in MeCN, at 25 °C, 50 mV s^−1^ and using iR compensation. EIS was carried out over the range 0.1–100 kHz using a 10 mV AC amplitude. Spectra were collected at either open circuit potential (OCP) or at varying DC offsets in 0.2 V steps as indicated in the text; 300 s equilibration time was allowed between each potential step. The specific capacitance was obtained *via* normalisation by the geometric area of the electrodes; this was determined in aqueous solution *via* a Randles–Sevcik plot,^14,20^ and in 0.1 M TBAPF_6_/MeCN *via* capacitance measurements on a reference GC disk of known area to account for any capillary wetting within the Teflon shielding in MeCN.^21^

## 3. Results and discussion

Nitrogenated carbon electrodes were prepared in the form of thin films *via* magnetron sputtering and characterised as described in previous work from our group.^14^ Briefly, introduction of N_2_ in the gas deposition at varying % flow ratios of 2%, 5% and 10% results in materials referred to as a-C:N-2%, a-C:N-5% and a-C:N-10%, respectively. Table 1 summarises XPS and Tauc gap results from previous work: the nitrogen content in the carbon scaffold increases with increasing N_2_ flow% and the increase is accompanied by an increase in semiconducting character, as indicated by Tauc gap values. All a-C:N surfaces have been shown to possess a mixture of pyridinic-N, pyrrolic-N and graphitic-N, and they were found to be smooth and conformal to the substrate surface.^14^

**Table 1:** Summary of properties of sputtered a-C:N electrodes used in our studies

Sample	N_2_%	N^a^ at%	N/C^a^ at%	Tauc gap^a^	*Φ* b (eV)
a-C	0%	n/a	n/a	0.66 ± 0.01	4.69 ± 0.03
a-C:N-2%	2%	8.3	15	0.25 ± 0.07	4.94 ± 0.02
a-C:N-5%	5%	15.6	28	0.19 ± 0.09	4.82 ± 0.01
a-C:N-10%	10%	19.5	35	0.7 ± 0.1	4.84 ± 0.02

a Values of N at% and Tauc gap determined *via* XPS and ellipsometry, respectively, from 14 and 16.

b Obtained from UPS in this work.

**Fig. 1:**
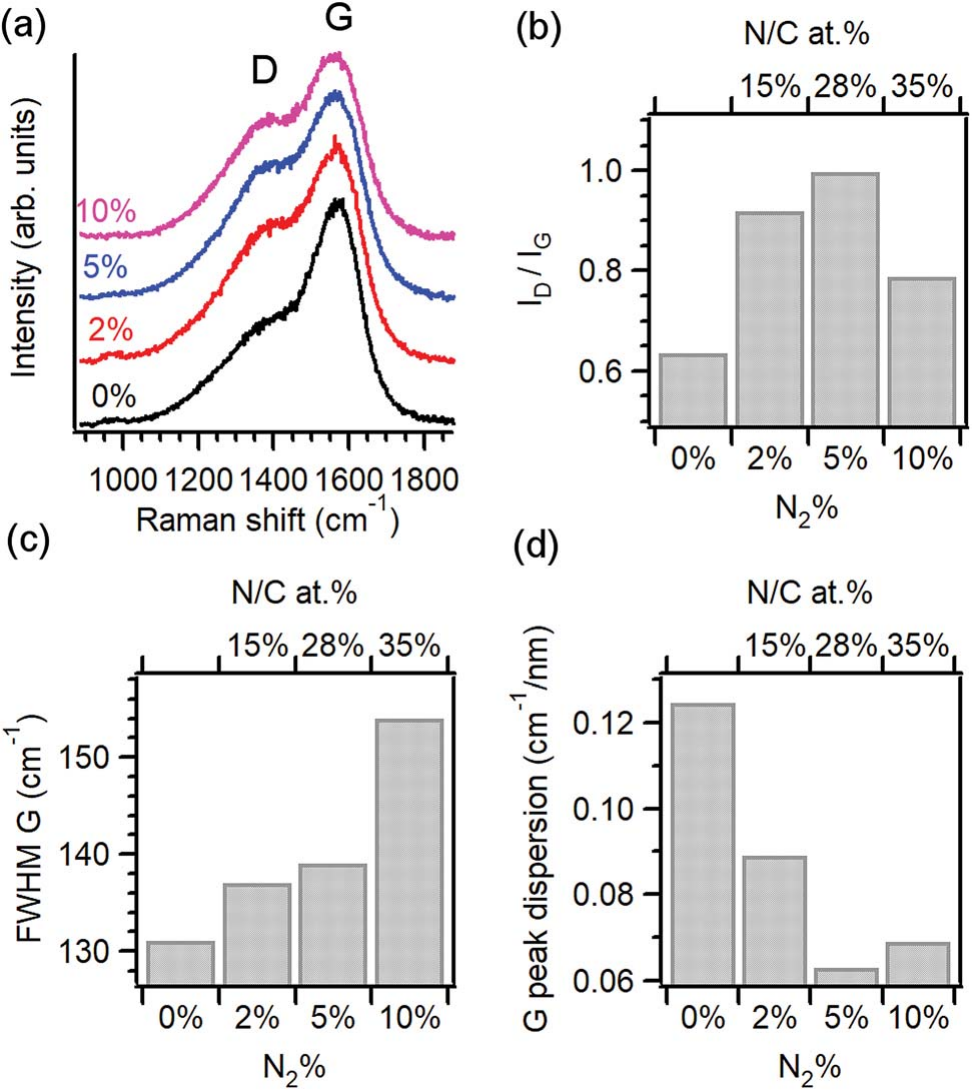
(a) Raman spectra of amorphous carbon electrodes prepared with varying N_2_% content in the deposition gas mixture: 0% (a-C), 2% (a-C:N-2%), 5% (a-C:N-5%) and 10% (a-C:N-10%); excitation 488 nm. Spectra are normalised relative to the G band intensity. (b) Variation of D to G peak height ratio (*I*_D_/*I*_G_), (c) G peak full width at half-maximum (FWHM) and (d) G peak dispersion *vs.* N_2_% content in the deposition gas (bottom axis) or *vs.* surface N/C% content (top axis).

Raman spectroscopy was used to characterise the structural properties of the amorphous carbon phase.^22–24^Fig. 1a shows baseline-corrected Raman spectra in the 900–1900 cm^−1^ range of a-C and a-C:N electrodes deposited on silicon wafers, obtained using 488 nm excitation. Spectra display two broad peaks characteristic of amorphous carbon materials, assigned to the G and D bands at approximately 1580 cm^−1^ and 1380 cm^−1^, respectively. The G band is associated with an optically allowed E_2g_ mode of sp^2^ centres, while the D band is associated with a disorder-allowed A_1g_ mode of six-membered carbon rings in graphite.^24–27^ Although it is not possible to exclude contributions from C–N and N–N modes in the same spectral region, Raman spectra of a-C:N materials is typically interpreted without an attempt to discriminate contributions from heterocyclic structures to these two main vibrational modes in the carbon matrix.^26^

The spectral profile changes significantly after the introduction of N_2_ in the deposition gas, as the D band increases in intensity relative to the G peak, suggesting a significant restructuring of the carbon matrix due to nitrogen incorporation. Spectra were deconvoluted using two Gaussian peaks^28–30^ and the main peak parameters were used to generate the data in Fig. 1b–d. Peak heights from best fits were used to calculate the *I*_D_/*I*_G_ ratio of each spectrum, which is diagnostic for amorphous carbon materials.^22,25,26,31,32^Fig. 1b shows the change in *I*_D_/*I*_G_*vs.* N_2_%-content in the deposition and its corresponding N/C concentration at the surface. Upon introduction of 2% N_2_ the relative height of the D band increases when compared with the nitrogen-free a-C material, which indicates an increase in the concentration of six-membered rings within the amorphous carbon scaffold.^26^ The *I*_D_/*I*_G_ ratio is known to increase as the average graphitic cluster size *L*_a_ increases in amorphous carbons, as discussed by Ferrari and Robertson;^25^ therefore, the increase of *I*_D_/*I*_G_ for a-C:N-2% relative to a-C confirms the important effect of nitrogenation on the clustering of sp^2^ centres. However, higher concentrations of N_2_ (>25% N/C at%) result in a decrease of the *I*_D_/*I*_G_ value, suggesting that at high concentrations, the dominant effect of these heteroatoms is that of disrupting the graphitic network in a-C:N, leading to greater defect density. This is supported by an analysis of the full width half maximum (FWHM) of the G band shown on Fig. 1c; this FWHM is diagnostic of the distribution of bond angles at excited sp^2^ centres and therefore tracks the local carbon disorder.^26^ It is evident from the figure that a slight increase in FWHM is observed for a-C:N-2% and −5% relative to a-C, whereas a-C:N-10% shows a major increase in FWHM that indicates a broad distribution of bonding geometries for sp^2^ centres.

The trends observed are in good agreement with those reported by Ferrari and co-workers for amorphous carbon films with increasing nitrogen content deposited using a variety of methods. Raman results for nitrogenated a-C:N films are generally more complex to interpret than for nitrogen-free a-C, due to the non-uniqueness of sp^3^-centre content and sp^2^ structuring that results from nitrogen incorporation.^26^ However, the dispersion of the G peak position is unequivocally associated with disordering as a result of nitrogenation; for this purpose, Raman spectra obtained at 633 nm excitation were also analysed to obtain the values of G-peak dispersion summarised in Fig. 1d. G peak dispersion falls sharply upon incorporation of nitrogen indicating an ordering effect resulting from nitrogenation.^25^ However, further nitrogen incorporation in a-C:N-10% does not result in greater ordering and a slight increase in dispersion is registered, in agreement with trends in Fig. 1b and c. In summary, analysis of Raman spectra indicates that a-C:N-2% and a-C:N-5% possess a more graphitic structure than nitrogen-free a-C; this is in agreement with previous determinations of Tauc gaps, which indicate an increase in metallic character for these two materials *vs.* a-C. Further incorporation of nitrogen to form a-C:N-10% however results in carbon materials that are significantly more defective, and that likely possess smaller graphitic cluster sizes. This confirms that using the range 0–10% N_2_ concentration in our deposition system it is possible to explore both the ordering and the defect-inducing effects of nitrogen incorporation on the electrochemical performance of non-crystalline carbon electrodes.

**Fig. 2:**
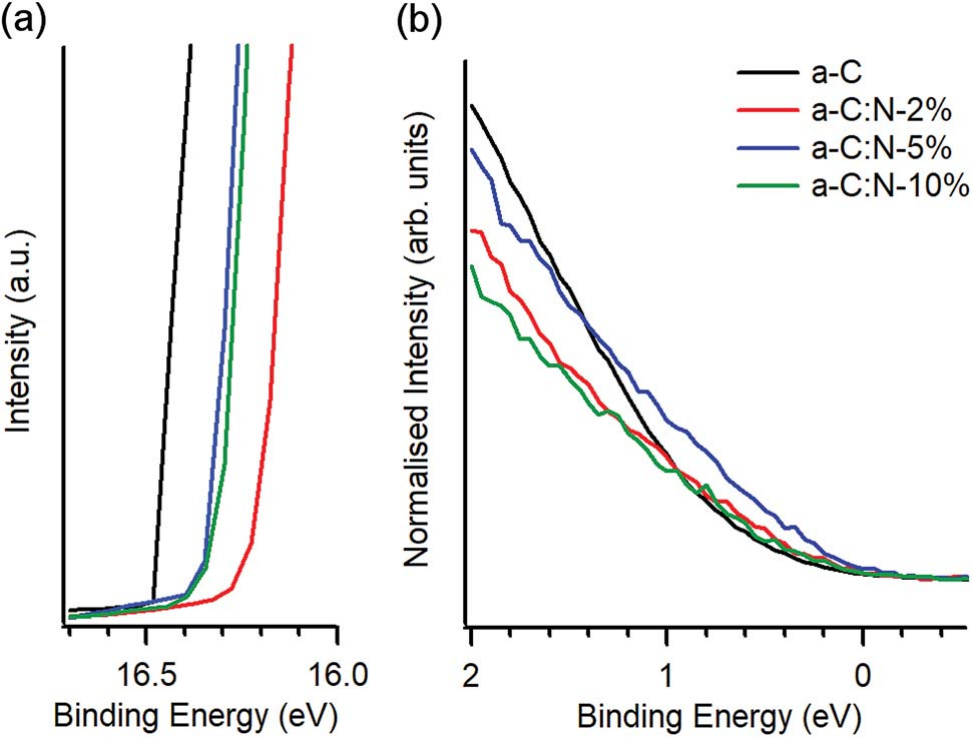
UPS of a-C and a-C:N electrodes. (a) High binding energy cutoff obtained at 10 V bias, showing the change in work function due to nitrogen incorporation. (b) Low binding energy region of N-free a-C and a-C:N materials showing photoemission near *E*_F_; spectra are shown normalised by the total photoemission intensity.

UPS was used to investigate the valence electronic properties of a-C:N electrodes. Fig. 2a and b show the UPS spectra in the high-binding and low-binding energy regions, respectively. The high binding energy edge was used to calculate work function values (*ϕ*), which are summarised in Table 1. The work function of a-C at 4.69 eV is in good agreement with previous reports on magnetron sputtered carbon^18,33^ and close to values quoted for graphitic nitrogen-free materials such as graphite (4.4 eV)^34,35^ and glassy carbon (4.6 eV).^35^ Incorporation of nitrogen results in an increase of work function values which fall in the range 4.82–4.94 eV; these values are above those of a-C but below those reported for O-terminated sputtered carbons (5.1 eV).^36^ The observed increase of *ϕ* upon N-modification is in agreement with experimental results by Wiggins-Camacho and Stevenson^12^ obtained from nitrogenated and N-free carbon nanotubes. Nitrogen incorporation can result in both an increase^12,37^ or a decrease^38,39^ in the work function of carbons arising from changes to semiconducting properties (*e.g.* n-type doping) and from the creation of surface functional groups. The observed increase suggests the presence of C–N terminations that add a positive contribution from surface dipoles to the work function,^18,37^ as observed for O-containing groups^36,38,40^ and as argued in the case of N-modified carbon nanotubes.^12^ No clear trend could be identified over the 2–10% deposition range, however it is possible that reorganisation of the carbon scaffold, *e.g.* due to defect creation^12^ or to clustering of sp^2^ centres,^26^ might further contribute to the net change in *ϕ*, thus resulting in a non-linear trend *vs.* N-content.

Fig. 2b shows details of the photoemission intensity of a-C:N samples and that of N-free a-C near *E*_F_. The intensity changes suggest that incorporation of nitrogen results in a slight increase in occupied states near the *E*_F_ for a-C:N-2% (N/C = 15 at%) and to a larger extent for a-C:N-5% (N/C = 28 at%); further incorporation in a-C:N-10% (N/C = 35 at%) appears to result in a decrease in occupied states. These observations are consistent with metallic/semiconducting character inferred from Tauc gap values (Table 1). Interestingly, the photoemission near *E*_F_ is maximised for a-C:N-5% which is also the sample that appears to be richest in graphitic clusters based on Raman results.

**Fig. 3:**
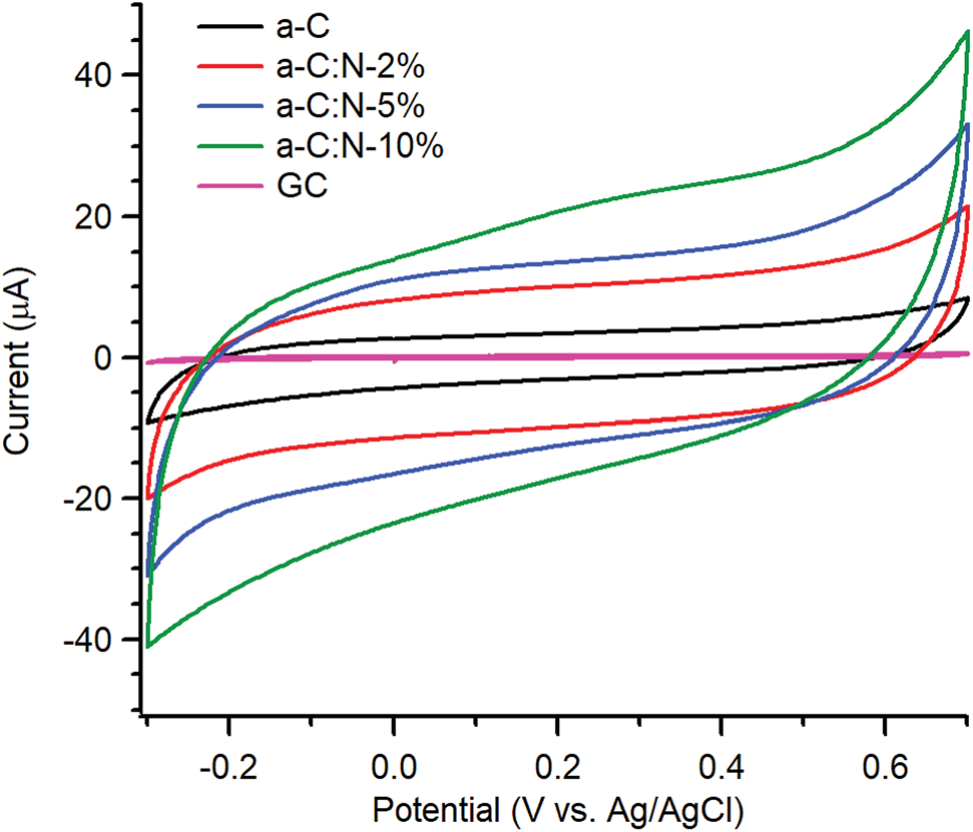
Cyclic voltammograms of GC, a-C, a-C:N-2%-10% in Ar-saturated 0.1 M KCl at 50 mV s^−1^.

Electrochemical characterisation *via* CV and EIS was carried out using a three-electrode system. Typical CVs of a-C and a-C:N electrodes over the −0.3–0.7 V potential window at 50 mV s^−1^ in 0.1 M KCl are shown in Fig. 3; the response of a polished GC substrate disk is included for comparison. The curves show the characteristic shape of a capacitive response, with all capacitive currents being larger than that of the GC substrate disk. Incorporation of nitrogen into the carbon scaffold leads to increased capacitance with progressively higher currents over a-C:N-2%-10%.

**Fig. 4:**
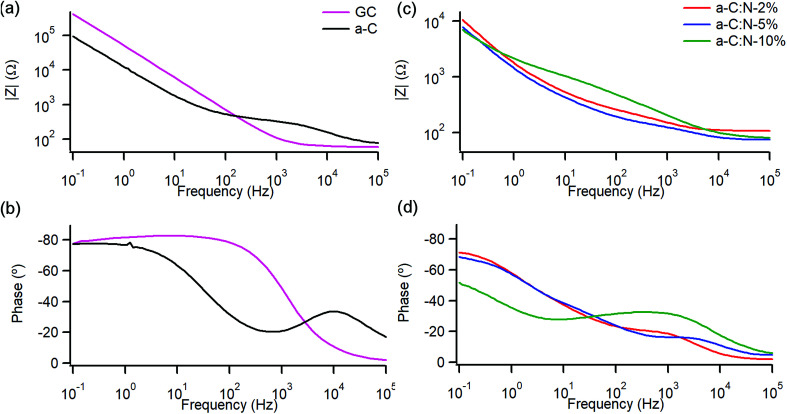
Bode plots of impedance module |*Z*| and phase angle of (a and b) nitrogen-free GC and a-C electrodes and (c and d) of nitrogenated a-C:N-2%, a-C:N-5% and a-C:N-10%. EIS spectra obtained in 0.1 M KCl at open circuit potential (OCP, 0.01–0.05 V *vs.* Ag/AgCl).

A study of the electrochemical response was carried out using EIS over the 0.1–10^5^ Hz range in the same solution. Fig. 4a and b show Bode plots of absolute impedance (|*Z*|) and phase angle obtained at OCP (0–0.05 V *vs.* Ag/AgCl), respectively, for nitrogen-free a-C electrodes and for GC as a reference graphitic electrode material. Results for the GC substrate are in good agreement with those reported for planar GC electrodes under similar conditions.^41^ The GC sample yields a response characteristic of an equivalent series RC circuit, thus consistent with a double layer capacitance (*C*_dl_) contribution with close to ideal behaviour. At 0.1 Hz the phase angle is approximately −83°, slightly below the ideal capacitor value of −90°, while at high frequency the response is resistive (phase ≈ 0°) with |*Z*| determined by the solution resistance (*R*_s_). a-C electrodes display a lower impedance at low frequency compared to that of GC and a phase angle of −77°, indicating a mainly capacitive response; however the appearance of an additional time constant evident from the phase plot at high frequency (∼600 Hz) suggests the presence of at least two distinct capacitive contributions. Fig. 4c and d show Bode plots obtained for a-C:N-2%-10% electrodes. The curves indicate that nitrogenation results in a further reduction in |*Z*| and greater deviations from ideal capacitive behaviour, while at high frequency the plots show evidence of additional capacitive contributions to the overall response.

The effective or equivalent series capacitive contribution to EIS spectra was calculated as a function of frequency *f* from the imaginary part of the complex impedance *Z*_im_ according to:

1 *C* = −(2π*fZ*_im_)^−1^

**Fig. 5:**
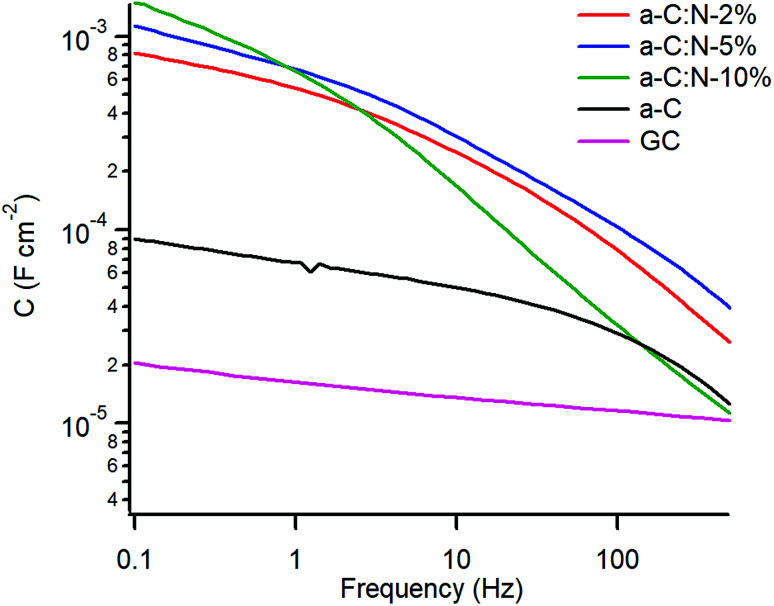
Equivalent series capacitance at OCP in 0.1 M KCl.

Fig. 5 shows a plot of the specific capacitance extracted over the 0.1–500 Hz range at OCP. The capacitance for the GC electrode was found to be 16 μF cm^−2^ at 1 Hz, in good agreement with reference values of *C*_dl_ in aqueous KCl,^20^ while the capacitance for nitrogen-free a-C is 4.4 times larger at *ca.* 70 μF cm^−2^. A very significant increase in capacitance is observed for a-C:N materials which yielded values in the mF cm^−2^ range. In the case of a-C:N samples there is also clear evidence of frequency dispersion, which is related to disorder and inhomogeneity in electrode surfaces,^42,43^ and this is seen to be particularly pronounced for a-C:N-10%.

**Fig. 6:**
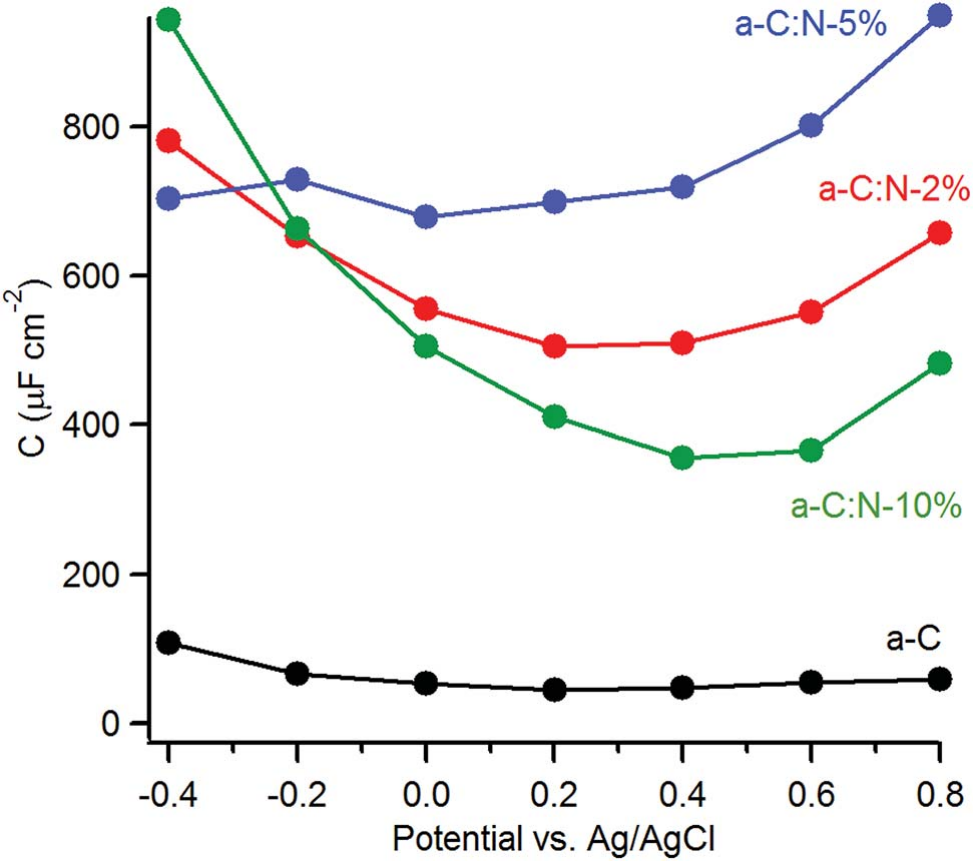
Equivalent series capacitance as a function of potential in 0.1 M KCl calculated at 1 Hz.

The capacitance of a-C:N materials as a function of potential was further investigated using EIS at varying DC offsets (see ESI†). Fig. 6 shows the change in the series equivalent capacitance at 1 Hz as a function of the DC offset over the range −0.4–0.8 V *vs.* Ag/AgCl in 0.1 M KCl. The nitrogen-free a-C electrode shows a shallow minimum in the capacitance that suggests a potential of zero charge (pzc) of 45 μF cm^−2^ at *ca.* 0.2 V. Nitrogenation leads to a considerable increase in the area-normalised capacitance. There is no detectable shift in the potential at the minimum capacitance for a-C:N-2% (*ca.* 0.5 mF cm^−2^) and 5% (*ca.* 0.7 mF cm^−2^), however, a significant positive shift is observed for a-C:N-10%, whose minimum (*ca.* 0.3 mF cm^−2^) falls in the range 0.4–0.6 V.

The capacitance values of nitrogenated a-C:N electrodes in Fig. 6 are large compared to those typical of planar carbon electrodes (1–70 μF cm^−2^ (ref. 20, 44 and 45)). This indicates that a-C:N materials possess intra-film capacitance due to porosity and/or pseudo-capacitive contributions. The presence of both such contributions is reasonable as a result of nitrogen incorporation. Previous work had shown that these a-C:N films are topographically smooth and featureless, however they might still allow for the presence of small cavities or “fissures” accessible to the electrolyte.^46^ As the capacitive response at low frequency results from probing by the AC signal deep into any pores present in the material,^47–49^ the large values observed could arise from intra-film porosity in all three a-C:N electrodes. This appears possible when considering the structurally disruptive effect of nitrogenation at high concentrations on the graphitic matrix.^14,26^ Beyond the development of a pore structure, nitrogenation can also introduce local surface states due to structural disorder in the carbon scaffold which are known to result in increased capacitance at low frequency.^46^ Finally, the presence of N-containing functional groups at the a-C:N surface in a protic solvent can promote specific adsorption^50^ and redox reactions at these sites (*e.g.* amine/hydroxylamine, amine/imine or pyridine/pyridone),^51–53^ thus introducing a pseudo-capacitive contribution in parallel to that of the double layer capacitance.

**Fig. 7:**
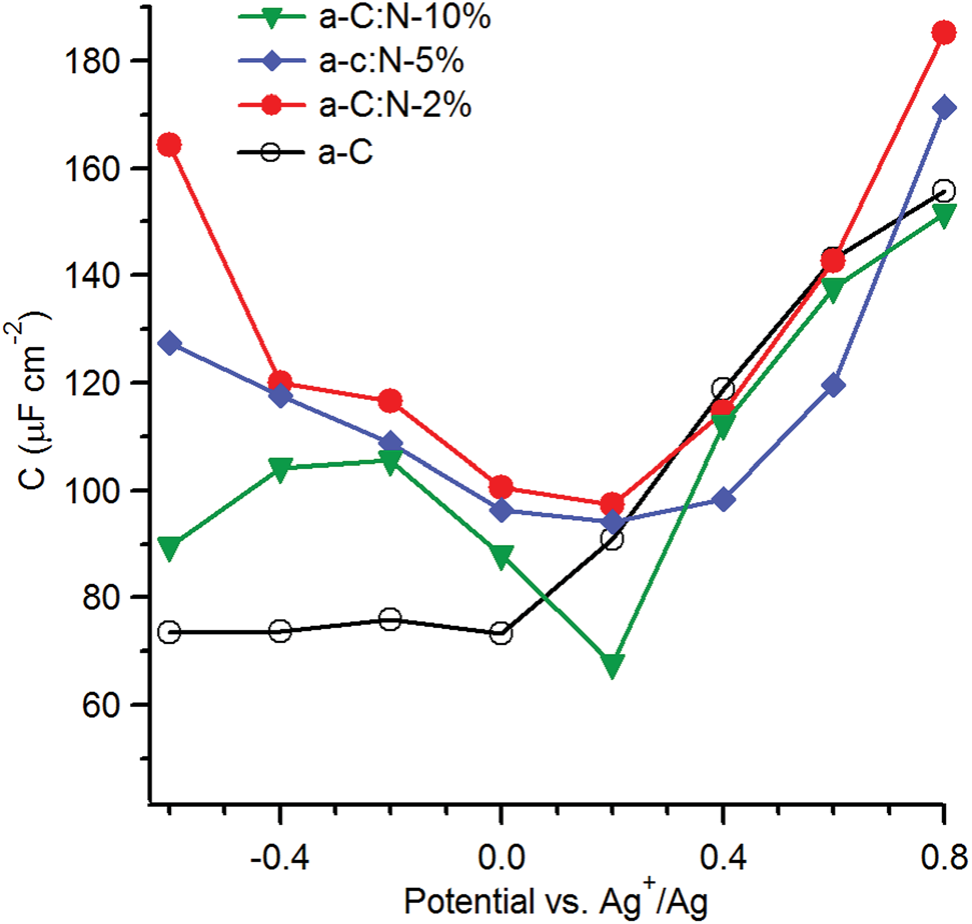
Equivalent series capacitance as a function of potential in 0.1 M TBAPF_6_ calculated at 1 Hz.

With the aim of investigating the effects of nitrogenation on the capacitive properties, while minimising complications arising from pseudo-capacitive effects, we carried out EIS studies in organic aprotic solvent using a 1 : 1 electrolyte with large ionic radii.^12,54,55^Fig. 7 shows the series equivalent capacitance at 1 Hz as a function of potential over the range −0.6–0.8 V *vs.* Ag^+^/Ag in 0.1 M TBAPF_6_ in acetonitrile for a-C and a-C:N electrodes. Cyclic voltammograms and representative EIS data over potentials close to the capacitance minima are reported in the ESI.† The capacitance curve of the a-C electrode is asymmetric with a capacitance at pzc of 73 μF cm^−2^ at 0 V *vs.* Ag^+^/Ag. This value is 3.5 times larger than that of a GC electrode disk measured under identical conditions (data not shown). The *C*_aC_/*C*_GC_ ratio is very close to that observed in aqueous electrolyte, therefore indicating that the larger capacitance of a-C electrodes relative to GC is mostly due to microroughness effects. Remarkably, while the capacitance at the potential of zero charge for the a-C:N samples in KCl are 5–10 times larger than that of a-C, the difference is instead small when the materials are tested in TBAPF_6_ solutions. The capacitances at pzc are remarkably close to each other and to the a-C value, being in a ratio *C*_aCN10_ : *C*_aC_ : *C*_aCN5_ : *C*_aCN2_ = 1 : 1.1 : 1.4 : 1.4. This strongly suggests that the large differences observed between a-C:N and a-C in KCl arise from pseudo-capacitive effects brought about by the presence of surface N-sites. Although it is not possible to exclude that intra-film porosity also contributes to the values in Fig. 5, protonation and faradaic activity of N-sites can be identified as the dominant contribution to the capacitive response of a-C:N electrodes in aqueous KCl.

The capacitance in TBAPF_6_/acetonitrile on the other hand is dominated by the double layer (*C*_dl_); furthermore, use of a high electrolyte concentration also ensures that the series contribution to the double layer capacitance arising from the diffuse layer (*C*_diff_) can be neglected. Under such conditions the observed capacitance is modulated by the electronic properties of a-C/a-C:N materials and by any differences in microroughness among the electrodes, thus enabling an analysis of the effects of nitrogen incorporation beyond those of surface chemical reactivity. The asymmetry in the a-C curve is consistent with an accumulation region at potentials anodic to 0 V *vs.* Ag^+^/Ag, which agrees with previous reports of p-type behaviour in nitrogen-free sputtered a-C.^18,56^ All of the plots for a-C:N materials display relatively symmetric minima at 0.2 V *vs.* Ag^+^/Ag, thus indicating that nitrogen incorporation into a-C results in an increase in the pzc.^57^ The shift in pzc relative to a-C is in good agreement with UPS data, which show that nitrogenation of a-C results in work function increases of 0.1–0.2 eV (Table 1). The minimum capacitance of a-C:N-10% is the lowest among a-C:N materials, while a-C:N-2% and a-C:N-5% display the largest ones; assuming that the microroughness factor remains constant across all sputtered electrodes, this finding is consistent with a-C:N-10% having the largest Tauc gap (Table 1) and greatest semiconducting character. The largest carrier density is achieved instead at low N/C contents.

The effects of nitrogen incorporation on the interfacial capacitance have been previously studied using carbon nanomaterials with long range order, such as graphene and nanotubes. Capacitance determinations in TBAPF_6_/acetonitrile of N-doped graphene with low N-content (<3 at%) show significant increases in capacitance as a result of doping. For instance, Jeong *et al.*^50^ observed a *ca.* 4-fold enhancement after N-doping *via* plasma treatment, while Zhang *et al.*^58^ reported a *ca.* 2-fold increase in the specific capacitance after N-doping during graphene growth. Interestingly, Zhu *et al.*^59^ investigated the effects of combined graphene N-doping (*ca.* 2%) and controlled defect introduction *via* Ar^+^ bombarding, achieving very significant increases in capacitance. In the case of carbon nanotubes, Wiggins-Camacho and Stevenson^12^ also reported a progressive enhancement of the *C*_pzc_ in TBAPF_6_/acetonitrile up to a factor of *ca.*3 for N-content <8 at%. In the case of our a-C:N materials the enhancement observed is comparatively modest (<40%), while for a-C:N-10% it is possible to actually observe a reduction in interfacial capacitance. This might be explained by the N-content being higher in our materials (15–35%) than in graphene/nanotube studies and by the concomitant presence of amorphous/non-crystalline regions. A higher N-content than 3–7% is frequently obtained for non-crystalline materials, and the data suggests that capacitive enhancements resulting from N-doping might be smaller once the N-content rises and/or the disorder increases. For instance, Zeng *et al.*^60,61^ observed marginal changes in the capacitance minimum of a-C:N electrodes deposited *via* filtered cathodic vacuum arc when N/C% increased from 8 to 17%, albeit in aqueous solutions. In TBAPF_6_/acetonitrile, Hulicova-Jurcakova *et al.*^62^ characterised the effect of N-content increases in porous nanocarbons and observed no increase in capacitance for N-content >10%. Literature results with non-crystalline materials support our observations; our previous work using redox couples and high N-content sputtered a-C:N electrodes^14^ showed that progressive inclusion of N-sites results in increased localisation of graphitic clusters within. It would therefore appear that capacitive enhancements can only be expected to result from N-doping when the nitrogenation process preserves a sufficient concentration of extended graphitic clusters in the carbon scaffold.

In the absence of an experimental determination of the microroughness factor for each electrode, it is not possible to obtain estimates of the density of states from values of *C*_pzc_, as previously done by other groups in the case of nanocarbon electrodes.^12,54^ However, it is interesting to note that *C*_pzc_ values are strongly correlated to the resistance to charge transfer (*R*_ct_) obtained in our previous work^14^ with the same electrode materials using Ru(NH_3_)_6_^+2/+3^, an outer-sphere redox couple, as shown in Table 2. The highest *R*_ct_ values correspond to the lowest *C*_pzc_ values; a similar trend is observed when the effective capacitance values are considered at −0.20 V, close to the formal potential of Ru(NH_3_)_6_^+2/+3^ (0.10 V *vs.* SHE,^19^ or −0.22 *vs.* Ag/Ag^+^). As the *R*_ct_ had been found to be inversely correlated to the density of states near the Fermi energy in these materials, this observation supports that the trends observed in Fig. 7 are controlled by the space-charge properties of a-C/a-C:N electrodes (albeit for small differences in microroughness). On the basis of the asymmetry observed in the curves, it is therefore possible to conclude that N-free a-C materials start as p-doped in character; a small amount of nitrogen incorporation as in a-C:N-2% has a drastic effect in reducing the p-character, as expected from the role of group V atoms as n-type donors. This is evident from the greater symmetry in the C *vs.* E curve and the increase in its *C*_pzc_ value, which are also consistent with the graphitizing effect of nitrogen incorporation observed from Raman. Further nitrogen incorporation, beyond ∼15 at% results in no apparent further increases in n-type character in the materials. This is likely due to rapid saturation of sites in the carbon matrix suitable for N-doping (graphitic-N); further increase in N/C at% involve the creation of functional groups that instead contribute to pseudocapacitance in aqueous solutions.

**Table 2:** Capacitance at potential of zero charge (*C*_pzc_) and comparison with resistance to charge transfer (*R*_ct_) reported^14^ for Ru(NH_3_)_6_^+2/+3^

Sample	*C*_pzc_ (μF cm^−2^)	*R*_ct_ (Ω)^14^
a-C	73	256
a-C:N-2%	97	46
a-C:N-5%	94	21
a-C:N-10%	67	197

## 4. Conclusion

In this work we have used sputtered thin films of amorphous carbon to investigate the effects of gradual nitrogen incorporation into the graphitic scaffold and its effects on the electronic and capacitive properties. The materials investigated varied in their total N/C content but all displayed a mixture of N-sites including pyridinic-N, pyrrolic-N, graphitic-N and N-oxides, and a combination of methods that are sensitive to both bulk and surface properties, was used for this purpose. Importantly, a comparison of capacitive storage of the same materials in aqueous and organic supporting electrolytes was carried out. Our results show that nitrogen incorporation significantly increases the capacitive storage in aqueous media relative to the N-free parent material. However, most of this increase can be attributed to pseudocapacitive contributions from redox-active N-sites. Measurements in organic electrolyte, which are dominated by the double layer capacitance, show that initial nitrogen incorporation into the disordered carbon scaffold compensates for p-type properties in the disordered carbon matrix, resulting in an increase in metallic character. Greater levels of nitrogenation, however, are disruptive and progressively increase the disorder and bandgap of the carbon material. It therefore appears that the effect of nitrogen as an n-type dopant is limited to low levels of nitrogenation that preserve graphitization in the carbon matrix, while higher N/C concentrations largely involve creation of defects and localised N-sites. This interpretation is consistent with bandgap results, work function and valence photoemission results. This combined experimental approach offers an effective strategy to discriminate between the local chemical effects of N-sites and those that impart a long-range effect on the metallic character of nitrogenated disordered carbon materials.

## Conflicts of interest

The authors declare no conflict of interest.

## Supplementary Material

RA-009-C8RA10187F-s001

## References

[cit1] Simon P., Gogotsi Y. (2013). Acc. Chem. Res..

[cit2] Wei L., Sevilla M., Fuertes A. B., Mokaya R., Yushin G. (2011). Adv. Energy Mater..

[cit3] Moussa G., Hajjar-Garreau S., Taberna P.-L., Simon P., Matei Ghimbeu C. (2018). C.

[cit4] Robertson J. (2002). Mater. Sci. Eng..

[cit5] Carbon Materials for Catalysis, ed. P. Serp and J. L. Figueiredo, John Wiley & Sons, Hoboken, New Jersey, 2009

[cit6] Properties of amorphous carbon, ed. S. R. P. Silva, INSPEC, Inc., The Institution of Electrical Engineers, London, 1st edn, 2003

[cit7] Guo D., Shibuya R., Akiba C., Saji S., Kondo T., Nakamura J. (2016). Science.

[cit8] Shao Y., Sui J., Yin G., Gao Y. (2008). Appl. Catal., B.

[cit9] Candelaria S. L., Shao Y., Zhou W., Li X., Xiao J., Zhang J.-G., Wang Y., Liu J., Li J., Cao G. (2012). Nano Energy.

[cit10] Deng Y., Xie Y., Zou K., Ji X. (2016). J. Mater. Chem. A.

[cit11] Latil S., Roche S., Mayou D., Charlier J.-C. (2004). Phys. Rev. Lett..

[cit12] Wiggins-Camacho J. D., Stevenson K. J. (2009). J. Phys. Chem. C.

[cit13] Zhou Y., Holme T., Berry J., Ohno T. R., Ginley D., O'Hayre R. (2010). J. Phys. Chem. C.

[cit14] Behan J. A., Stamatin S. N., Hoque M. K., Ciapetti G., Zen F., Esteban-Tejeda L., Colavita P. E. (2017). J. Phys. Chem. C.

[cit15] Cullen R. J., Jayasundara D. R., Soldi L., Cheng J. J., Dufaure G., Colavita P. E. (2012). Chem. Mater..

[cit16] Zen F., Karanikolas V. D., Behan J. A., Andersson J., Ciapetti G., Bradley A. L., Colavita P. E. (2017). Langmuir.

[cit17] Zen F., Angione M. D., Behan J. A., Cullen R. J., Duff T., Vasconcelos J. M., Scanlan E. M., Colavita P. E. (2016). Sci. Rep..

[cit18] Colavita P. E., Sun B., Tse K.-Y., Hamers R. J. (2007). J. Am. Chem. Soc..

[cit19] Kerr J. and Lide D., CRC Handbook of Chemistry and Physics, 2000

[cit20] Kissinger P. and Heineman W. R., Laboratory Techniques in Electroanalytical Chemistry, Taylor & Francis, 2nd edn, revised and expanded, 1996

[cit21] De Levie R. (1965). J. Electroanal. Chem..

[cit22] Chu P. K., Li L. (2006). Mater. Chem. Phys..

[cit23] Ferrari A. C., Robertson J. (2004). Philos. Trans. R. Soc. London, A.

[cit24] Ferrari A. C., Rodil S. E., Robertson J. (2003). Diamond Relat. Mater..

[cit25] Ferrari A. C., Robertson J. (2000). Phys. Rev. B.

[cit26] Ferrari A. C., Rodil S. E., Robertson J. (2003). Phys. Rev. B.

[cit27] Gilkes K. W. R., Prawer S., Nugent K. W., Robertson J., Sands H. S., Lifshitz Y., Shi X. (2000). J. Appl. Phys..

[cit28] Nathan M. I., Jr J. E. S., Tu K. N. (1974). J. Appl. Phys..

[cit29] Robertson J. (1992). Surf. Coat. Technol..

[cit30] Tai F. C., Lee S. C., Chen J., Wei C., Chang S. H. (2009). J. Raman Spectrosc..

[cit31] Tamor M. A., Vassell W. C. (1994). J. Appl. Phys..

[cit32] Yoshikawa M., Katagiri G., Ishida H., Ishitani A., Akamatsu T. (1988). J. Appl. Phys..

[cit33] Murphy D. M., Cullen R. J., Jayasundara D. R., Doyle R. L., Lyons M. E. G., Colavita P. E. (2013). J. Phys. Chem. C.

[cit34] Chen E. C. M. and Chen E. S. D., in The Electron Capture Detector and the Study of Reactions with Thermal Electrons, John Wiley & Sons, Inc., 2004, pp. 47–74

[cit35] Lipkin H. J. (1949). Phys. Rev..

[cit36] Colavita P. E., Sun B., Wang X., Hamers R. J. (2009). J. Phys. Chem. C.

[cit37] Kaukonen M., Nieminen R. M., Pöykkö S., Seitsonen A. P. (1999). Phys. Rev. Lett..

[cit38] Yang N., Yang D., Chen L., Liu D., Cai M., Fan X. (2017). Nanoscale Res. Lett..

[cit39] Xu J., Mei J., Huang X. H., Li X., Li Z., Li W., Chen K. (2005). Appl. Phys. A.

[cit40] Ago H., Kugler T., Cacialli F., Salaneck W. R., Shaffer M. S. P., Windle A. H., Friend R. H. (1999). J. Phys. Chem. B.

[cit41] Metz K. M., Colavita P. E., Tse K.-Y., Hamers R. J. (2012). J. Power Sources.

[cit42] Pajkossy T. (1994). J. Electroanal. Chem..

[cit43] Kerner Z., Pajkossy T. (2000). Electrochim. Acta.

[cit44] Fagan D. T., Hu I. F., Kuwana T. (1985). Anal. Chem..

[cit45] Ranganathan S., Kuo T.-C., McCreery R. L. (1999). Anal. Chem..

[cit46] Randin J.-P., Yeager E. (1975). J. Electroanal. Chem. Interfacial Electrochem..

[cit47] Fletcher S., Black V. J., Kirkpatrick I. (2014). J. Solid State Electrochem..

[cit48] Yoo H. D., Jang J. H., Ryu J. H., Park Y., Oh S. M. (2014). J. Power Sources.

[cit49] Lufrano F., Staiti P. (2004). Electrochem. Solid-State Lett..

[cit50] Jeong H. M., Lee J. W., Shin W. H., Choi Y. J., Shin H. J., Kang J. K., Choi J. W. (2011). Nano Lett..

[cit51] Kim J.-I., Park S.-J. (2011). J. Solid State Chem..

[cit52] Béguin F., Szostak K., Lota G., Frackowiak E. (2005). Adv. Mater. (Weinheim, Ger.).

[cit53] Wang D.-W., Li F., Yin L.-C., Lu X., Chen Z.-G., Gentle I. R., Lu G. Q., Cheng H.-M. (2012). Chem. – Eur. J..

[cit54] Gerischer H., McIntyre R., Scherson D., Storck W. (1987). J. Phys. Chem..

[cit55] Hahn M., Baertschi M., Barbieri O., Sauter J.-C., Kötz R., Gallay R. (2004). Electrochem. Solid-State Lett..

[cit56] Hastas N. A., Dimitriadis C. A., Panayiotatos Y., Tassis D. H., Patsalas P., Logothetidis S. (2000). J. Appl. Phys..

[cit57] Trasatti S. and Parsons R., in Pure Appl. Chem., 1986, vol. 58, p. 437

[cit58] Zhang L. L., Zhao X., Ji H., Stoller M. D., Lai L., Murali S., McDonnell S., Cleveger B., Wallace R. M., Ruoff R. S. (2012). Energy Environ. Sci..

[cit59] Zhu J., Childress A. S., Karakaya M., Dandeliya S., Srivastava A., Lin Y., Rao A. M., Podila R. (2016). Adv. Mater. (Weinheim, Ger.).

[cit60] Zeng A., Bilek M. M. M., McKenzie D. R., Lay P. A. (2009). Diamond Relat. Mater..

[cit61] Zeng A., Neto V. F., Gracio J. J., Fan Q. H. (2014). Diamond Relat. Mater..

[cit62] Hulicova-Jurcakova D., Kodama M., Shiraishi S., Hatori H., Zhu Z. H., Lu G. Q. (2009). Adv. Funct. Mater..

