# Supplementary material for: Capacitive storage at nitrogen doped amorphous carbon electrodes: structural and chemical effects of nitrogen incorporation
Source: RSC Adv. 2019 Jan 30;9(7):4063–71. doi: 10.1039/c8ra10187f (PMC9060499; doi:10.1039/c8ra10187f)
Supplement: RA-009-C8RA10187F-s001 [file RA-009-C8RA10187F-s001.pdf]

Electronic Supplementary Material (ESI) for RSC Advances.
This journal is © The Royal Society of Chemistry 2019

Supporting Information

Capacitive Storage at Nitrogen Doped Amorphous Carbon Electrodes: Structural and Chemical Effects of Nitrogen Incorporation

Md. Khairul Hoque,[a] James A. Behan,[a] Serban N. Stamatin,[a,b] Federico Zen,[a] Tatiana S. Perova[c] and Paula E. Colavita[a1]

a – School of Chemistry, CRANN and AMBER Research Centres, Trinity College Dublin, Dublin 2, Ireland

b – University of Bucharest, Faculty of Physics, 3Nano-SAE Research Centre, 405 Atomistilor Str., Bucharest-Magurele 077125, Romania

c – Department of Electronic and Electrical Engineering, Trinity College Dublin, Dublin 2, Ireland and ITMO University, 49 Kronverskiy pr., Saint Petersburg, 197101, Russia.

[1] Corresponding author: colavitp@tcd.ie

S1. Thickness determinations for a-C and a-C:N thin film electrodes

The thickness of deposited films was determined using spectroscopic ellipsometry according to previously reported protocols.[1-3] Briefly, the a-C or a-C:N electrode was modelled as a 3-layer structure incorporating the underlying Si wafer substrate, native oxide layer and thin film. The complex index of refraction and thickness of the carbon layer were parameterized using B-Splines which have previously been used to accurately determine bulk optical properties and film thicknesses on amorphous carbon materials.[4] The resulting thicknesses in each case were normalised by the constant deposition time of 40 minutes to obtain a deposition rate in nm min^{-1} as a function of the N_2% in the sputtering chamber as reported in Figure S1.

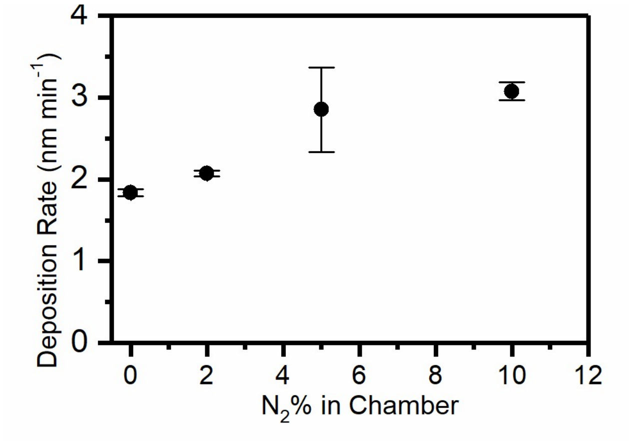

Figure S1. Deposition rate obtained from spectroscopic ellipsometry determinations for a-C[1] and a-C:N materials[3] as a function of N_2% flow in the deposition chamber under conditions identical to those of our experiments.

S2. Nyquist plots in aqueous 0.1 M KCl

Figure S2 shows Nyquist plots of the a-C and a-C:N electrodes whose capacitance is reported in Figure 6 in the main text; the plots show data at three potentials near the capacitance minima.

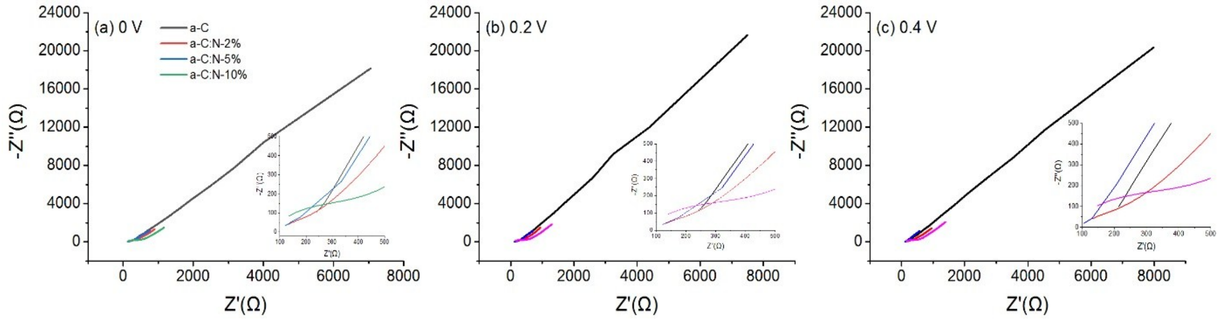

Figure S2. Nyquist plots of EIS spectra of a-C, a-C:N-2%, a-C:N-5%, a-C:N-10% in aqueous 0.1 M KCl at (a) 0.0 V, (b) 0.2 V and (c) 0.4 V vs. Ag/AgCl.

S3. Cyclic voltammograms in 0.1 M TBAPF

Figure S3 shows cyclic voltammograms of a-C and a-C:N electrodes in 0.1 M $TBAPF_6$ in acetonitrile at varying scan rate.

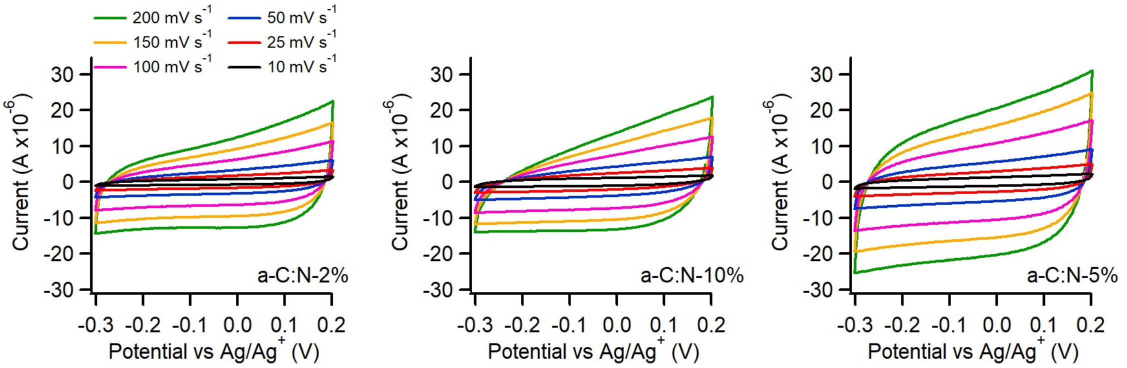

Figure S3. Cyclic voltammograms of a-C:N-2%, a-C:N-5% and a-C:N-10% electrode in 0.1 M $TBAPF_6$/acetonitrile at varying scan rates. No Faradaic peaks are visible in the potential window used for our experiments.

S4. Nyquist plots in aqueous 0.1 M $TBAPF_6$

Figure S4 shows Nyquist plots of the a-C and a-C:N electrodes whose capacitance is reported in Figure 7 in the main text; the plots show data at three potentials near the capacitance minima.

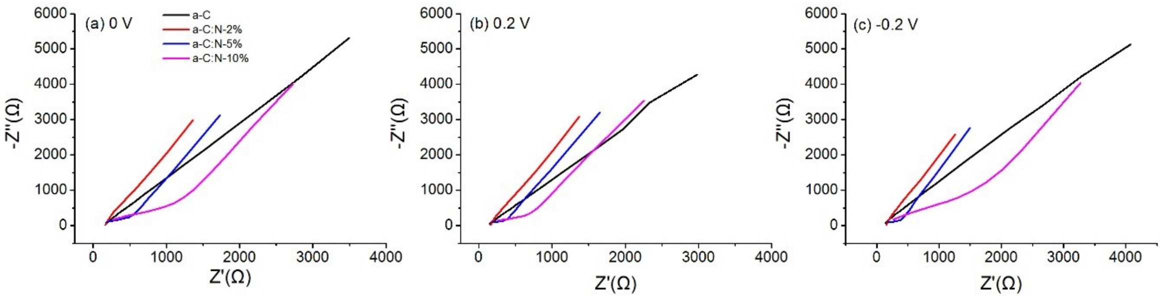

Figure S4. Nyquist plots of EIS spectra of a-C, a-C:N-2%, a-C:N-5%, a-C:N-10% in 0.1 M $TBAPF_6$ in acetonitrile at (a) 0 V, (b) 0.2 V and (c) -0.2 V vs. Ag^+/Ag.

References

1. Zen, F.; Karanikolas, V. D.; Behan, J. A.; Andersson, J.; Ciapetti, G.; Bradley, A. L.; Colavita, P. E., Nanoplasmonic Sensing at the Carbon-Bio Interface: Study of Protein Adsorption at Graphitic and Hydrogenated Carbon Surfaces. *Langmuir* **2017,** *33*, 4198-4206.

2. Zen, F.; Angione, M. D.; Behan, J. A.; Cullen, R. J.; Duff, T.; Vasconcelos, J. M.; Scanlan, E. M.; Colavita, P. E., Modulation of Protein Fouling and Interfacial Properties at Carbon Surfaces via Immobilization of Glycans Using Aryldiazonium Chemistry. *Sci. Rep.* **2016,** *6*, 24840.

3. Behan, J. A.; Stamatin, S. N.; Hoque, M. K.; Ciapetti, G.; Zen, F.; Esteban-Tejeda, L.; Colavita, P. E., Combined Optoelectronic and Electrochemical Study of Nitrogenated Carbon Electrodes. *J. Phys. Chem. C* **2017,** *121*, 6596-6604.

4. Weber, J. W.; Hansen, T. A. R.; van de Sanden, M. C. M.; Engeln, R., B-spline parametrization of the dielectric function applied to spectroscopic ellipsometry on amorphous carbon. *J. Appl. Phys.* **2009,** *106*, 123503.
